# Determining the Prevalence and Seasonality of *Fasciola hepatica* in Pasture-based Dairy herds in Ireland using a Bulk Tank Milk ELISA

**DOI:** 10.1186/s13620-015-0042-5

**Published:** 2015-07-09

**Authors:** Yris Bloemhoff, Andrew Forbes, Martin Danaher, Barbara Good, Eric Morgan, Grace Mulcahy, Mary Sekiya, Ríona Sayers

**Affiliations:** Animal and Bioscience Research Department, Animal & Grassland Research and Innovation Centre, Teagasc, Moorepark, Fermoy, Co. Cork, Republic of Ireland; Merial, 29 Avenue Tony Garnier, 69007 Lyon, France; Food Safety Department, Food Research Centre, Ashtown, Teagasc, Dublin 15, Republic of Ireland; Animal and Grassland Research and Innovation Centre, Teagasc, Athenry, Co, Galway, Republic of Ireland; School of Biological Sciences, University of Bristol, Woodland Road, Bristol, BS8 1UG UK; School of Veterinary Medicine, University College Dublin, Belfield, Dublin 4, Republic of Ireland

**Keywords:** Fasciola hepatica, Epidemiology, Dairy cows, ELISA, Seasonality

## Abstract

**Background:**

*Fasciola hepatica* is a helminth parasite of global importance in livestock, with major economic impact. However information on *F. hepatica* infections in Irish pasture-based dairy herds is limited. Therefore this study was conducted in order to determine the prevalence, seasonality and management factors associated with *F. hepatica*. A total of 319 Irish dairy herds were selected for this study. Bulk tank milk (BTM) samples were collected from 290 dairy farms on a quarter year basis, while from a further 29 dairy farms BTM samples were collected on a monthly basis to provide a more detailed pattern of *F. hepatica* exposure in Irish herds. BTM samples were analysed using a commercially available *F. hepatica* antibody detection ELISA. Furthermore, within-herd prevalence of *F. hepatica* was assessed in a subset of these 29 herds (n = 17); both individual serum samples and bulk tank milk samples were collected.

**Results:**

A within-herd prevalence of ≤ 50 % was found for herds with negative bulk tank milk samples. The mean prevalence of the 290 study herds was 75.4 % (Range 52 %–75.1 %), with the highest prevalence being observed in November (75.1 %). The seasonal pattern of *F. hepatica* shows elevated antibodies as the grazing season progressed, reaching a peak in January. A significant association was found between *F. hepatica* and age at first calving.

**Conclusion:**

This study demonstrates that *F. hepatica* is present in a large proportion of Irish dairy herds and provides a basis on which control practices, particularly in adult dairy cows, can be reviewed.

## Background

The trematode *Fasciola hepatica* (liver fluke) is an important helminth parasite in livestock worldwide. The lifecycle of *F. hepatica*, similar to other trematodes, involves both a final (e.g. cattle, sheep) and an intermediate snail (*Galba truncatula*) host. *F. hepatica* outbreaks have a seasonal pattern with two waves of infection in summer and winter [1]. Infections with *F. hepatica* in livestock can result in significant economic losses, from decreased productivity, liver condemnations and mortality [2]. More specifically, in dairy cows and breeding heifers, these infections often remain subclinical, but result in reduced milk productivity and fertility [3].

Diagnosis of *F. hepatica* was previously based on coprological techniques alone, but with the advent of coproantigen, copro-PCR and enzyme linked immunosorbent assays (ELISA) for the diagnosis of *F. hepatica* in sera and milk, detection of *F. hepatica* has become more sensitive [4, 5]. The use of ELISA on bulk tank milk (BTM) samples has allowed monitoring of an entire herd for *F. hepatica* status, and–when applied to individual milk samples-determination of the within-herd prevalence of *F. hepatica* [5, 6]. Currently, control of *F. hepatica* is generally achieved using anthelmintic treatment, however more widespread application of herd ELISA status data will allow greater evidence-based control of *F. hepatica* at farm level [6]. This in turn will contribute to more appropriate and sustainable use of flukicide treatments in dairy herds [7], which could result in a reduction in anthelmintic use and reduce selection for anthelmintic resistance [8].

Previous international studies have been conducted to document the prevalence of *F. hepatica* infection in a number of Western European countries. The prevalence ranged from 37.3 % in Flanders, Belgium, to 76 % in the UK [9, 10]. An Irish abattoir study reported that *F. hepatica* was present in 65 % of the livers of culled dairy and beef cows [11]. In a more recent study by Selemetas et al. [12] a prevalence of 67 % was found. The high prevalence of fluke in Irish dairy cows is not surprising given that Irish climatic conditions include abundant rainfall and lack of temperature extremes. This favours both the survival of *F. hepatica* and its intermediate host *G. truncatula* [13]. Additionally, the vast majority of Irish dairy farmers operate pasture-based seasonal-calving milk production systems [14] with the majority of cows calving during a compact period in spring from February to April [15], thereby maximizing milk production from low-cost grazed grass [16]. In general, Irish dairy cows are grazed outdoors on pasture from as early as February until as late as December, when weather conditions allow [17]. Irish cattle therefore have greater potential for exposure to and infection with *F. hepatica* compared to cattle reared in different livestock systems and climatic conditions. As only limited studies regarding the prevalence of *F. hepatica* infections exist in Ireland and heretofore no nationally representative study has been completed, the primary objective of this study was to determine the prevalence of *F. hepatica* in a geographically representative group of Irish dairy herds. Secondary objectives included determination of the within herd prevalence detectable by a specific bulk milk ELISA kit, and also investigation of the usefulness of this kit in highlighting seasonal patterns of liver fluke infestation in Irish herds.

## Methods

### Prevalence Study–selection of farms and sample collection

Dairy herds were selected from the HerdPlus® database containing 3,500 members, which represented 18 % of the Irish national dairy population in 2009. HerdPlus® contains records from dairy herds and is a breeding information decision support tool coordinated by the Irish Cattle Breeding Federation (ICBF). To yield sufficient study power a total of 500 dairy farms were randomly selected from HerdPlus®, with the prospect of yielding 300 dairy farmers. To join the ‘HerdAhead’ program a stratified sampling procedure based on herd size and geographical location was applied to select HerdPlus® dairy farms. The study population has previously been shown to geographically represent the Irish dairy farm population O’Doherty et al. [18]. A total of 312 farms volunteered to participate in the study, resulting in study sample size that yielded a 95 % confidence level and 5 % confidence interval based on a herd prevalence of 70 % (i.e. there is 95 % confidence that the results generated in the current study are representative of the national population of dairy herds).

In 2009, the bulk of these farms (n = 290 ‘Herdhead’) were asked to submit a BTM sample on a quarterly year basis (23rd March, 8th June, 31st August, 2nd November), while 22 herds were selected to participate in a monthly BTM sampling programme. The 22 herds were selected on the basis that the farmers were members of the Dairy Management Information System (‘DairyMIS’) discussion group coordinated by Teagasc (Irish Agriculture and Food Development authority). These commercial farms were located in Munster in the south-west of Ireland. An additional 7 Teagasc herds were included in this sub-group called ‘DairyMIS’.

BTM samples were collected using a standardized kit, which has previously been described in detail by O’Doherty et al. [18]. Briefly, this sampling kit contained a 500 ml jug, a 250 ml sampling container containing five milk preservative tablets (Broad spectrum Microtabs 2, D & F Control systems inc., USA), a submission form, sampling instructions and a cover letter informing the farmer of the required sampling date. To remind the dairy farmers of the sampling date a text message was forwarded the day prior to and on the day of sampling. The acquired bulk tank milk sample was then returned by express post with all samples received within 48 hours of sampling.

Between 2010 and 2012 BTM samples continued to be collected from the ‘DairyMIS’ herds (n = 29) using a slightly modified sampling kit. The sample kit contained a 50 ml sample bottle (Sarstedt, Germany), which contained a Broad Spectrum Microtab milk preservative tablet (D&F Control systems inc., USA) a submission form, sampling instructions and a cover letter informing the farmer of the required sampling date. The sampling dates were generally planned on the day of the ‘DairyMIS’ meeting on the first Wednesday of the month, allowing farmers to hand in the samples, otherwise express post was used for rapid delivery. On arrival to the laboratory, bulk tank milk samples were aliquoted into duplicate 2.5 ml micro tubes (Sarstedt, Germany), centrifuged at 20,000 g for 1 minute, de-fatted and the supernatant transferred to 1.5 ml microtubes (Sarstedt, Germany) and frozen at–80 °C until further analysis.

### Within herd prevalence: study population: selection of farms and sample collection

A subset of these ‘DairyMIS’ herds referred to as ‘Dairy17’ herds (n = 7 in 2010 and n = 10 in 2012, with 5 herds collected in both years), were chosen to examine within-herd prevalence of *F. hepatica*. Herds were chosen on the basis of ELISA results from BTM samples which represent negative, low positive, moderate positive and high positive bulk tank milk readings. All milking cows were blood sampled by coccygeal venepuncture using a standard 18 gauge needle into plain vacutainers with no anticoagulant, within 15 days (mean = 9 days) of the bulk milk sample collection. Blood samples were centrifuged at 3000 rpm for 3 min within 12 hours of collection. Serum was aliquoted into 1.5 ml microtubes and frozen at–20 °C degrees until further use.

### Sample preparation and ELISA

*F. hepatica* analysis was completed using a commercially available ELISA kit, with a sensitivity [Se] and specificity [Sp] of 98 % (Ildana Biotech, Dublin, Ireland). All tests were carried out “in-house” according to kit manufacturer’s instructions and previously described in detail by Selemetas et al. [12]. The assay is based on a recombinant mutant *F. hepatica* cathepsin L1 antigen (CL1) [19], which is produced as an inactive enzyme in *Pichia pastoris*. Plates are coated with 0.01 mg/ml antigen in carbonate coating buffer in alternate columns on a 96-well ELISA plate (EIA/RIA stripwell plates, Sigma-Aldrich, St, Louis, MO, USA) leaving each alternate strip uncoated. Samples were done in duplicate. Positive and negative bovine serum controls were supplied with the kit and used for S/P determination. The optical density (OD) reading of the uncoated well was subtracted from the reading of the coated well to yield a corrected OD. The ratio of the sample OD to the positive control OD was subsequently calculated to yield the S/P ratio. Likewise antibodies in serum were analysed using the same *F. hepatica* ELISA kit. A 1:20 ratio of bovine sera to sample buffer was used, 190 μl of sample buffer was mixed with 10 μl of control serum in each well.

A positive cut-off of 15 and 20 S/P was used for BTM and serum samples respectively.

### Management and herd classification

A questionnaire was used to collect management data from ‘HerdAhead’ farms and has previously been described by Bloemhoff et al., [20]. Briefly farmers supplied information regarding general farm management, dosing and grazing management of cows, in-calf heifers, and calves (Table 1). Additionally herd size was downloaded from the ICBF database.

**Table 1:** Independent variables of ‘HerdAhead’ farms per category, with independent variables used in the multivariate model

Independent variables	Categories		Independent variables
		% (n)	
Region	Region 1	21.7 (60)	Region 1 vs. Region 2 vs. Region 3
	Region 2	30.3 (84)	
	Region 3	48.0 (133)	
Herd size	≤50	9.4 (26)	<50 cows vs. 50–100 cows vs. >100 cows
	50-100	50.4 (139)	
	≥100	40.2 (111)	
Herd enterprise	Dairy only	47.5 (131)	Dairy only vs. Mixed enterprise
	Mixed	52.5 (145)	
Calving period	Spring	85.5 (236)	Spring-calving vs. Mixed-calving
	Mixed	14.5 (40)	
Number of 1st lactation heifers	<25	60.7 (168)	<25 heifers vs. 25–50 heifers vs. >50 heifers
	25-50	32.9 (91)	
	>50	6.5 (18)	
Age heifers 1st calving	<24 months	10.2 (27)	<24 months vs. 24–30 months vs. >30 months
	24-30 months	82.6 (219)	
	>30 months	7.2 (19)	
Flukicide treatment cows	Not treated	34.3 (95)	Not treated vs. Treated
	Treatment for flukicides	65.7 (182)	
Flukicide treatment heifers	Not treated	31.8 (88)	Not treated vs. Treated
	Treatment for flukicides	68.2 (189)	
Turnout cows	Jan/Feb	83.6 (230)	January/February vs.
	Mar/Apr	16.36 (45)	March/April
	Other	-	Excluded
Housing cows	Sep/Oct	28.1 (75)	September/October vs. November/December
	Nov/Dec	71.9 (192)	
	Other		Excluded
Stocking rate cows	<5 cows	87.7 (236)	Less than 5 cows vs. 5-10/ >10 cows
	5-10 cows />10 cows	12.3 (33)	
Grazing length cows	<7 months	32.2 (86)	<7 months grazing vs. >7 months grazing
	>7 months	67.8 (181)	

**Fig. 1:**
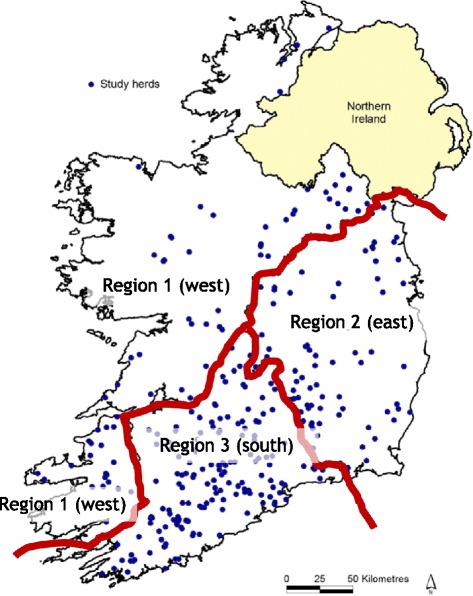
Map of the Republic of Ireland with study herds devided into three regions

Study farms were allocated to three regions, described as Region-1 (West; counties, Donegal, Monaghan, Cavan, Longford, Leitrim, Sligo, Roscommon, Mayo, Galway, Clare, Kerry), Region-2 (East; counties, Louth, Meath, Westmeath, Kildare, Dublin, Offaly, Laois, Carlow, Wicklow, Wexford, Kilkenny) and Region-3 (South; counties, Tipperary, Limerick, Waterford, Cork) and outlined in Fig. 1. The map was created using ESRI Arcview 3.2 (Redlands, California, USA). The location of study herds was attributed to the centroid of the largest fragment of land for each herd according to the Land Parcel Identification System (LPIS) for 2008 (Fig. 1). The calving period of study herds was split into two categories i.e. spring-calving (i.e. majority of the herd calved between January and April) and mixed-calving (i.e. a proportion of the herd calved between January and April with remaining cows calved at other times of year). Livestock enterprise on the study herds was divided into two categories, namely dairy only (only dairy animals on the farm) or mixed enterprise (dairy plus beef and/or sheep). Flukicide treatment of adult cows, in-calf heifers and calves was previously described by Bloemhoff et al. [20], as was grazing season length.

**Fig. 2:**
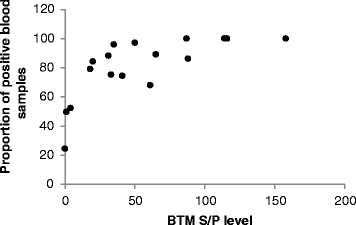
The within-prevalence of ‘Dairy17’ herds showing the correlation between positive individual blood S/P values and corresponding mean positive BTM herd samples

**Fig. 3:**
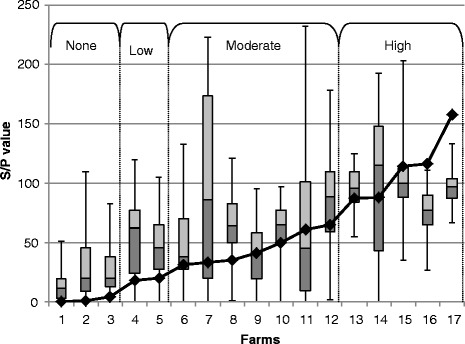
Increasing bulk tank milk S/P results (Line) and corresponding S/P values of individual blood samples within each herd, showing the minimum, 25th percentile, median, 75th percentile and maximum for each of the ‘Dairy17’ herds

### Data analysis

Descriptive analysis was performed using PROC FREQ and PROC UNIVARATE (SAS version 9.3, USA). Graphical representations were generated and Student *T*-Test using Excel (Version MS Office 2003). Univariable and multivariable generalized estimating equation (GEE) analysis was completed using PROC GENMOD (SAS, version 9.3, USA).

The apparent prevalence (Ap) of ‘HerdAhead’ herds was calculated for each sampling date for *F. hepatica* (Table 2). The overall annual prevalence for ‘HerdAhead’ herds is tabulated by recording at least one positive result of *F. hepatica* at any one sampling date. The true prevalence (Tp) for each herd was calculated using Rogan Gladen Epitools [21]. The seasonal trend in both Ap and Tp was tabulated. In addition, the distribution of low positive, moderate positive and high positive at each sampling point was completed. Student *T*-test using a one tailed distribution with two sample unequal variance, was used to compare the proportion of *F. hepatica* positive ‘HerdAhead’ herds in each Region (-1,-2,-3) at each sampling date (Table 3, Fig. 1).

**Table 2:** Apparent (Ap) and true prevalence (Tp) of *F. hepatica* for ‘HerdAhead’ herds

	Apparent prevalence	True prevalence	
	%	%	CI*
March	52.0	52.1	45.9-58.2
June	53.8	53.9	47.8-60.1
August	62.5	63.0	57.0-68.9
November	75.1	76.1	70.8-81.4

*95 % Confidence interval (CI)

**Table 3:** Percentage of *F. hepatica* positive ‘HerdAhead’ herds in each region at each sampling date and overall percentage positive

	Region 1	Region 2	Region 3
March	60 %	48.8 %	50.4 %
June	61.7 %	50 %	52.6 %
August	73.3 %	60.7 %	58.7 %
November	86.7 %	77.4 %	68.4 %
Overall	70.4 %	59.2 %	57.5 %

As a first step a univariable analysis was performed using binomial dependent variable *F. hepatica* bulk tank milk result and independent variables, with herd included as a repeated measure. A binomial distribution was assumed and a logit link function used. An exchangeable correlation was applied for the analysis. Independent variables significant at a p < 0.10 in the univariable analysis were included in the multivariable model. A manual backward regression with a forward step was performed to build the final model. Two-way interactions deemed biologically significant were included in the analysis and retained in the final model at a significance level of p < 0.05.

## Results

The herds are a national representative sample spread across three regions (West, East and South) (Fig. 1). Due to missing data, 13 ‘HerdAhead’ herds were excluded, leaving 277 ‘HerdAhead’ herds for statistical analysis.

### General management

All of the study herds had full access to pasture throughout most of the year (average 8 months, range 5–10 months). The mean herd size of study herds was 97.5 cows (range 28 to 400 cows). February (n = 170, 74.6 %) was the month when the majority of farmers turned out their adult cows, while housing was mostly performed in November (n = 137, 60.1 %). Close to 50 % of the study herds had a livestock enterprise of dairy only. An average of 24.5 first lactation heifers, were added to the adult herds (range 3–150) with an age of approximately 24.9 months (range 22–36 months).

### Within-herd prevalence study

The ‘Dairy17’ herds were used to evaluate the relationship between the proportion of positive blood samples and the matching bulk tank milk S/P value (Fig. 2). In addition the individual blood samples of each herd were plotted in a boxplot, with attached bulk tank milk sample S/P result (Fig. 3). Each of the herds was categorized relative to the bulk tank milk result (Negative/None, low positive, moderate positive and high positive). The within-herd prevalence of *F. hepatica* of the ‘Dairy17’ herds was calculated as the number of individual cows positive at the >20 S/P value as a percentage of all cows serum sampled. Both Figs. 2 and 3 show a good agreement between the BTM S/P range and the percentage of cows positive to *F. hepatica*. The three herds that had a negative BTM result had less than half of the cows’ serum samples positive to *F. hepatica*. In addition these negative BTM herds had considerably lower mean S/P values (S/P = 21), compared to positive BTM herds (S/P = 73). There was a good correlation between BTM S/P results and the individual blood S/P results in the subset of BTM positive herds (Fig. 3). BTM positive herds had a mean within-herd prevalence of approximately 88 % of cows seropositive to *F. hepatica*.

### Prevalence

**Fig. 4:**
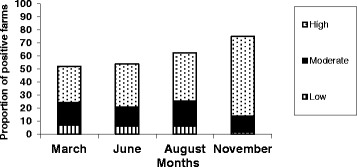
Within each month HerdAhead herds (n = 277) are categorised based on S/P value into low positive, moderate positive and high positive S/P herds

Results on prevalence from the ‘HerdAhead herds (n = 277) are shown in Table 2 and Fig. 4 respectively. 12 farms were omitted from the study because of missing data. Positive herds were characterized as low (15–25 S/P), moderate (25–75 S/P), and highly (>75 S/P) positive for *F. hepatica*. A total of 75.4 % of the ‘HerdAhead’ herds recorded one or more positive *F. hepatica* BTM results across the four sampling dates. The November sampling point recorded the highest proportion of positive ‘HerdAhead’ herds and herds with a BTM status of ‘High positive’. Multivariable analysis revealed similar results to the prevalence data (Table 4). Herds were more likely to be positive in November compared to March (OR = 2.74), June (OR = 2.62) and August (OR = 1.80), while herds in August were more likely to be positive to *F. hepatica* compared to March (OR = 1.53) and June (OR = 1.46).

**Table 4:** Multivariate model of *F. hepatica* to dependent variables

Variables in model (model p value)	Direction dependent variable	Explanation	Odds ratio	Confidence interval (95 %)	p-value
Age heifers first calving (p = 0.0582)	Positive vs. Negative	24-30 months vs. <24 months	1.64	0.82, 3.29	0.1630
		>30 months vs. <24 months	3.46	1.15, 10.41	0.0269
		>30 months vs. 24–30 months	2.11	0.84, 5.31	0.1120
Sampling date (p = <.0001)		June vs. March	1.05	0.90, 1.22	0.5482
		August vs. March	1.53	1.28, 1.82	<.0001
		November vs. March	2.74	2.16, 3.48	<.0001
		August vs. June	1.46	1.24, 1.72	<.0001
		November vs. June	2.62	2.08, 3.30	<.0001
		November vs. August	1.80	1.49, 2.17	<.0001

### Seasonality

To investigate the seasonality of *F. hepatica* for each farm the percentage of herds recording BTM values positive (P= > 15 S/P) and negative (n = <15 S/P) were tabulated. For example, a farm recording a negative result in March and June and positive results in August and November, this would be described as ‘nnPP’. The positive/negative time course for ‘HerdAhead’ herds is shown in Table 5. Close to half of the study herds had four positive results to *F. hepatica*. In general study herds were positive to *F. hepatica* towards the end of the grazing season in November. Approximately 8 % of study farms had three positive results out of four sampling dates, of which all had a positive result in November. Approximately 8 % of the herds had two positive results, of which 97 % were positive in November. A total of 34 farms had one positive result in November, whereas 19 farms had two positive results namely in August and November.

**Table 5:** Proportion of HerdAhead herds (n = 277) in each category of antibody status (P = above threshold, n = below threshold) at each sampling time point for *F. hepatica*

March	June	August	November	%	n
P	P	P	P	47.6	132
n	n	n	n	24.5	68
P	n	n	n	0.4	1
n	n	n	P	10.8	30
P	n	n	P	0.7	2
n	P	n	P	0.7	2
n	n	P	P	6.9	19
P	P	n	P	0.4	1
P	n	P	P	2.9	8
n	P	P	P	5.1	14

**Fig. 5:**
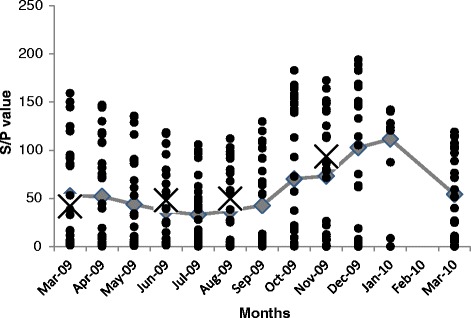
Seasonality of antibodies to *F. hepatica* on 29 DairyMIS farms and HerdAhead for between March 2009 and March 2010; line tracks the mean S/P

Monthly bulk milk samples from 2009 of the ‘DairyMIS’ herds (n = 29) were used to evaluate the seasonality. Monthly bulk milk sample S/P values were plotted for each herd, with the mean S/P value of all 29 farms also calculated and plotted (Fig. 5). The seasonality of *F. hepatica* infections in ‘DairyMIS’ herds was also calculated. A reduced number of herds were sampled in December, January and February, as cows were in the dry period. The majority of ‘DairyMIS’ herds were spring-calving herds (Calving cows in spring), and dry-off their dairy cows in November or December. The monthly seasonality of *F. hepatica* infection is shown in Fig. 5 for ‘DairyMIS’ and highlights similar results to ‘HerdAhead’ study herds. The seasonal pattern generally remained steady over the March to August 2009 months. However from September on the mean S/P values of ‘DairyMIS’ farms increased until the peak in January. In addition during the winter period the mean S/P values decreased after January and settled in March 2010 at similar values to March 2009.

### Multivariable analysis

The results of the multivariable analyses are shown in Table 4. Herds with heifers calving over 30 months of age were more likely (OR = 3.46) to be positive to *F. hepatica* compared to herds with heifers calving <24 months (p = 0.0269). No statistical regional differences were found in the multivariable analysis, however region was found to be significant in the univariable analysis. Table 3 shows that the proportion of herds positive to *F. hepatica* in Region–1 is higher than Regions–2 and–3. However the Student *T*-test did not find a significant difference between Regions–1 vs.-2 (p = 0.075) and–2 vs.-3 (p = 0.393), although there was a significant difference between Region 1 vs. 3 (p = 0.0296).

## Discussion

This study was used to determine the prevalence of *F. hepatica* in Irish dairy herds using a BTM *F. hepatica* ELISA. This study revealed that a large proportion (75 %) of the ‘HerdAhead’ study herds were positive to *F. hepatica* across the 2009 lactation. This is slightly higher than previously reported in an Irish abattoir study, which found that 65 % of the livers of culled dairy and beef cows were infected, but is consistent with a recent study carried out by Selemetas et al. [12], which used similar BTM ELISA methodology. In many countries in mainland Europe, *F. hepatica* prevalence is considerably lower [3, 9, 10, 22]. In two abattoir studies a prevalence of 18 % and 28 % were found in Switzerland and in regions in Portugal and Spain, respectively [3, 22]. Moreover in studies using bulk tank milk a prevalence of 37 % to *F. hepatica* was found in Belgium, while in Germany a prevalence of 23.6 % was found in adult cows [23, 24]. However in the United Kingdom, the prevalence of *F. hepatica* (76 %) based on BTM samples was similar to that found in this study [2]. Ireland and the UK have a similar climate, which is predominantly influenced by the Atlantic gulf stream [25], which can result in abundant rain, moisture and limited temperature ranges that are favourable for the development and survival of both *F. hepatica* and its intermediate host *Galba truncatula* [26]. Moreover the management system used on many Irish dairy farms is mostly pasture-based. The majority of Irish dairy herds graze pastures fulltime for up to 10 months of the year, with grass comprising around 70 % of the diet [27]. These management factors have previously been found to be directly linked to the exposure to metacercariae [23]. The late housing (up to December) of dairy cows could also contribute to the exposure to *F. hepatica* as pasture infectivity is the highest during the autumn [23]. The UK study also established regional differences between *F. hepatica* prevalence [2]. In the current study, a significant difference was found between Region–1 (West) and–3 (South). This is not surprising given that the soil type is generally quite different between the two regions, with Region-1 containing a higher proportion of less well-drained soil compared to Region–3, where soil types are less heavy. It has also shown that farmers in Region–1 are more likely to use flukicides and anthelmintics for the treatment of *F. hepatica* [20] which suggests that these farmers in Region–1 are aware of *F. hepatica* as a problem in their area.

The seasonal pattern found in this study shows a rise in September towards the end of the grazing season, which has previously been described in literature [23, 28]. The late season rise in September could be explained due to increased infected snails in summer, which infect the pasture with metacercariae in summer/autumn. Cows start picking up metacercariae around May or June, which could explain the increase of antibodies from August onwards. When these herds are housed and dried off, these antibodies generally drop. This might be explained due to the lack of metacercariae exposure during the dry period when spring-calving dairy cows are generally housed, and control of *F. hepatica* infection with an anthelmintic can be achieved [6]. However, from an epidemiological point of view, treatment might be more advantageously administered in August. However, due to the lack of availability of appropriate control measures for lactating dairy cows, treatment can often be difficult or impractical [11]. The limited number of flukicides available for dairy cattle all have withdrawal periods, which makes it more difficult to be used during lactation as valuable milk has to be discarded [23]. In addition the majority of flukicides available for dairy cows can only treat mature *F. hepatica* and therefore a subsequent treatment is required. To adhere to withdrawal periods the main time for flukicide treatments is during the dry period for spring-calving herds [7], which in Ireland coincides with housing during November until February in the majority of the herds. In autumn-calving herds, which are lactating during this housing period, treatment during the dry period would be in summer, but at this time of year, cows are grazing and can pick up new *F. hepatica* infections. Therefore treatment with flukicides in these herds might not be optimal. This study highlights the importance of implementing adequate *F. hepatica* control strategies across the island of Ireland. In addition, approximately 50 % of the herds had four positive results to *F. hepatica*, suggesting that significant production losses due to *F. hepatica* in about half of Irish dairy herds are likely, further emphasizing the need to implement adequate control-strategies in Irish dairy herds. Losses due to *F. hepatica* have been estimated to be around €299 per infected cow, due to reduced milk yield, compromised reproductive performance, liver condemnations and reduced meat production [3, 10, 29]. Moreover *F. hepatica* infections can reduce the reproductive performance and life-time milk production of heifers. In-calf heifers calving around 2 years of age have a considerable advantage over heifers that calve later, as they are more likely to produce more milk and calves over their productive lifetime [30]. In this study, heifers that calved over 30 months of age were more likely to be in a herd positive for *F. hepatica*, compared to heifers that calved at 24 months and younger. Although reproductive performance in high-input high-output systems is a very important factor, it is of more importance in seasonal calving systems. This is to maintain a compact calving pattern. Ideally breeding of the majority of the cows in such systems should be achieved within a 6 week breeding period in May and June [31]. While management and nutritional factors are the main determinants of reproductive performance in Irish dairy herds, optimal performance may also be hampered by *F. hepatica* infections during the breeding period. In this study, over half of the dairy herds were positive to *F. hepatica* during the crucial breeding month of June [31]. Therefore more research should be performed on the production losses due to *F. hepatica* infections in heifers and dairy cows.

The ELISA clearly showed seasonal changes in bulk-milk *F. hepatica* antibodies. In addition there was a good correlation between the negative BTM results and the within-herd prevalence of *F. hepatica* positive animals in these herds. Therefore, the *F. hepatica* ELISA is a useful and easy to use tool for farmers and veterinarians.

## Conclusion

 This study shows a high prevalence of *F. hepatica* in Irish dairy herds at the end of the grazing season, but also a considerable amount during the grazing season. There is a need for appropriate control measures in adult dairy cows, especially during the lactation. Additionally the Ildana ELISA showed clearly the seasonal changes of *F. hepatica* and is therefore a useful and easy to use diagnostic tool.

## Abbreviations

Ap: Apparent prevalence
BTM: Bulk tank milk
Cl1: Cathepsin L1 antigen
ELISA: Enzyme linked immunosorbent assay
ICBF: Irish cattle breeding federation
n: Negative
OD: Optical density
OR: Odds ratio
P: Positive
PCR: Polymerase chain reaction
S/P: Sample positive
Tp: True prevalence
